# Fluid–structure interactions (*FSI*) based study of low-density lipoproteins (*LDL*) uptake in the left coronary artery

**DOI:** 10.1038/s41598-021-84155-3

**Published:** 2021-02-26

**Authors:** Xueping Chen, Jian Zhuang, Huanlei Huang, Yueheng Wu

**Affiliations:** 1 Institute of Biomechanics, School of Bioscience and Bioengineering, South China University of Technology, Guangzhou, 510006 People’s Republic of China; 2 Department of Cardiovascular Surgery, Guangdong Cardiovascular Institute, Guangdong Provincial Key Laboratory of South China Structural Heart Disease, Guangdong Provincial People’s Hospital & Guangdong Academy of Medical Sciences, School of Medicine, South China University of Technology, Guangzhou, 510080 People’s Republic of China

**Keywords:** Biomedical engineering, Computational biology and bioinformatics

## Abstract

The purpose of this study is to compare the effect of the different physical factors on low-density lipoproteins (*LDL*) accumulation from flowing blood to the arterial wall of the left coronary arteries. The three-dimensional (3D) computational model of the left coronary arterial tree is reconstructed from a patient-specific computed tomography angiography (*CTA*) image. The endothelium of the coronary artery is represented by a shear stress dependent three-pore model. Fluid–structure interaction ($FSI$) based numerical method is used to study the *LDL* transport from vascular lumen into the arterial wall. The results show that the high elastic property of the arterial wall decreases the complexity of the local flow field in the coronary bifurcation system. The places of high levels of *LDL* uptake coincide with the regions of low wall shear stress. In addition, hypertension promotes *LDL* uptake from flowing blood in the arterial wall, while the thickened arterial wall decreases this process. The present computer strategy combining the methods of coronary *CTA* image 3D reconstruction, $FSI$ simulation, and three-pore modeling was illustrated to be effective on the simulation of the distribution and the uptake of *LDL*. This may have great potential for the early prediction of the local atherosclerosis lesion in the human left coronary artery.

## Introduction

According to the report from the American Heart Association (AHA), coronary atherosclerosis accounts for over 30% of cardiovascular diseases^1^. Previous studies suggested that atherosclerosis lesion regions usually held local complex hemodynamic forces, such as vortex and oscillatory shear^2^. Hemodynamic studies found that the blood flow is always complex around the arterial bifurcations where atherosclerosis lesions usually occur^3^. Many pieces of evidence suggested that the initiation and progression of the atherosclerotic disease involves a significant accumulation of low-density lipoproteins (*LDL*) in the arterial wall^4,5^. Due to the high clinical incidence of coronary atherosclerosis, it is of great clinical significance to study the distribution of hemodynamic parameters and the accumulation of *LDL* in the coronary artery.

Previous studies revealed that atherosclerotic plaque predisposed area coincides with the regions of high LDL concentration distribution^6,7^. However, concentration distribution alone cannot explain the focal accumulation of *LDL* within the arterial wall. Endothelium is a major barrier of the *LDL* transport from the artery lumen into the vessel wall^8^. There are two pathways for *LDL* via the endothelium: (1) vesicles absorb *LDL* from plasma by receptor-mediated endocytosis; (2) *LDL* directly pass through endothelium by leaky junctions^9^. Early experimental studies have shown that more than 90% of *LDL* transport into the arterial wall through leaky junctions while only less than 10% of *LDL* flux via vesicular pathway^10^. However, the leaky junctions-based pathway is associated with endothelial cells in the state of mitosis or apoptosis^11^. Researches showed that the fraction of leaky junction on endothelium is influenced by the shear stress on the luminal surface^12^.

The arterial wall included multi-layers in physiologically. It was widely known that the distribution of the flow filed in arteries is co-regulated by all layers of the wall. Moreover, the LDL transport in the flowing blood is controlled by the convection–diffusion equation. To study the LDL transport to the arterial wall, previous researchers proposed many models for the representation of the multi-layer arterial wall, such as the four-layer model and the single-layered model^13–17^. However, these studies were based on the CAD-generated models that were unphysiological. In the current work, we want to go further than the existing literature on predicting the distribution and the uptake of LDL in the coronary arterial wall. To predict the *LDL* accumulation in the arterial wall, we introduced a three-pore model to represent the endothelium^11,18^. This model is fully taking account of the contribution of the vesicular pathway, normal junctions, and leaky junctions to the transport of *LDL* from lumen to the arterial wall. According to the three-pore model, *LDL* transport was greatly governed by the local wall shear stress ($WSS$)^12^, therefore, it was of great clinical significance to figure out which factors may be the key cause of disturbing the blood flow. Moreover, the numerical model was generated from a patient-specific CTA image, which made the study model closer to physiological conditions. Complex vascular geometric structure and pulsatile could lead to a spatial and temporal alterations of blood flow in the human arterial system^19^. This made $WSS$ difficult to measure in vivo. Fortunately, fluid–structure interactions ($FSI$) technique is capable of capturing arterial wall move and flow change with high time and spatial resolution. In this study, two-way fully coupled $FSI$ simulations were carried out to capture the real-time changes of the intravascular flow field. The numerical simulations were performed with ANSYS 14.0 software. Hemodynamic parameters such as time-averaged wall shear stress ($TAWSS$), oscillatory shear index ($OSI$), relative residence time ($RRT$) and time-averaged wall shear stress gradient ($TAWSSG$) were analyzed in the present study^20–22^.

## Methods

### Reconstruction of left coronary artery models

The *CTA* image used in this study came from a patient by medical examination in the Guangdong General Hospital. The voxel size of the coronary CTA image was 0.5 × 0.5 × 0.5 mm. This study was approved by the Research Ethics Committee of the Guangdong General Hospital, Guangdong Academy of Medical Sciences and conformed to the principles outlined in the Declaration of Helsinki.

**Figure 1:**
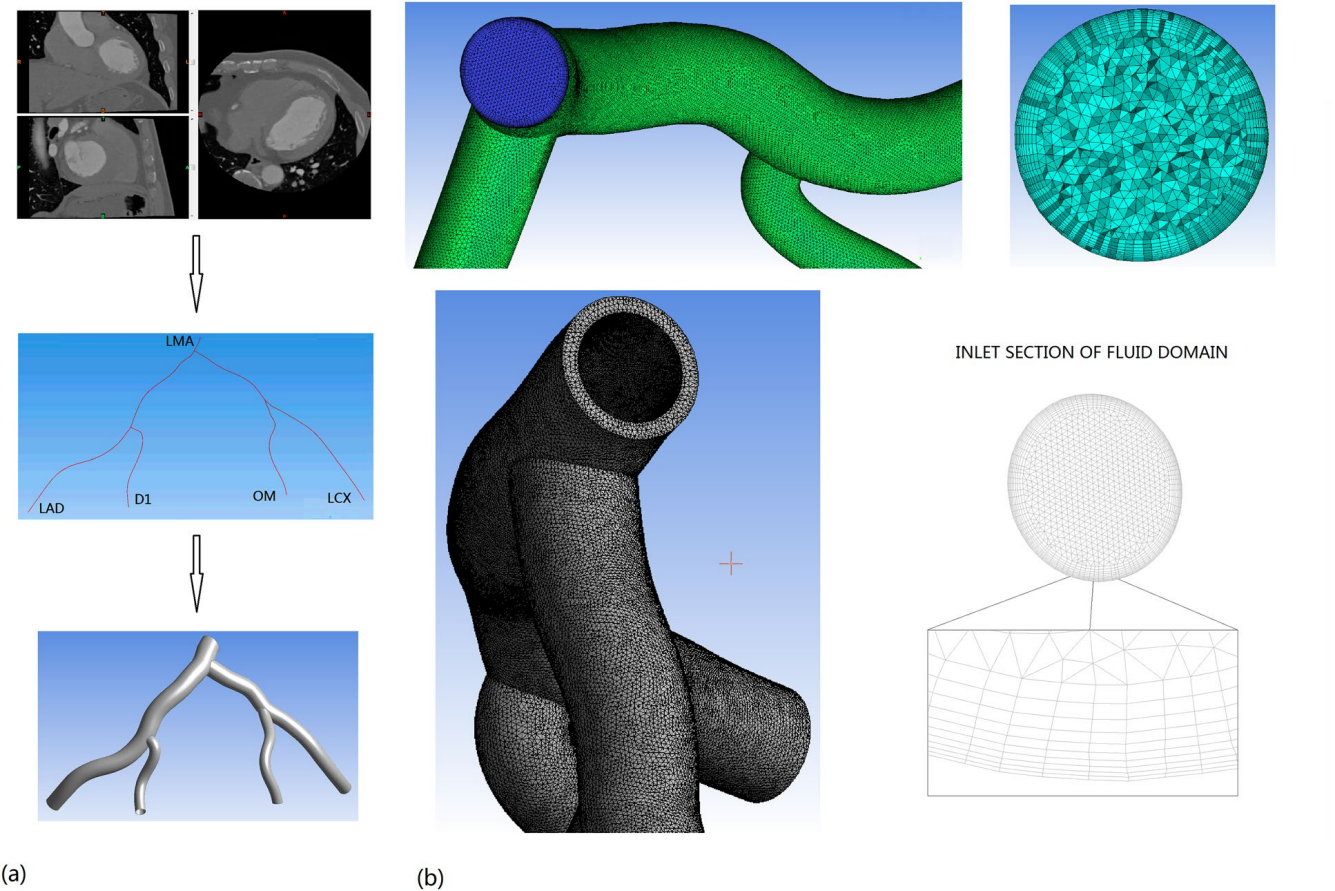
Reconstruction of 3D human left coronary artery. (**a**) Reconstructed human left coronary artery from the original *CT* coronary data of a 54-year-old male patient. *LMA* left main coronary artery, *LAD* left anterior descending artery, *LCX* left circumflex artery, *D1* first diagonal branch of the *LAD*, *OM* left obtuse marginal branch. (**b**) Meshes of blood-phase & arterial wall of human left coronary artery.

Many good results were analyzed with the use of Computer-Aided Design (CAD)-generated arteries in the previous studies^23,24^. However, these geometric models deviated from the physiological characteristics to a certain extent. In this study, the 3D fluid domain, as shown in Fig. 1a, was generated from the *CTA* image of a patient-specific left coronary artery. The first step was to extract and refine the centerline of the human left coronary arteries with the use of MIMICS software. The centerlines were formed by a series of center points that are located at the center of the cross-sectional plane of the 3D coronary artery. Then proceed as follows, dividing along the centerline to obtain circle borders of the human left coronary arteries on the vertical plane of the centerline, sweeping the circle borders, guided by the centerlines, to obtain the 3D model of the human left coronary artery. Lastly, we meshed the reconstructed model with the use of ANSYS ICEM-CFD software.

A typical structure of an arterial wall includes six layers. These layers are glycocalyx, endothelium, intima, internal elastic lamina, media, and adventitia, respectively. To simplify the numerical model in this study, the arterial wall was regarded as a single-layer with constant thickness. In our present study, the coronary arterial wall was given the thicknesses of $WTH=0.53\,\text{  mm}$ and $WTH=0.77\text{  mm}$ for the normal and thickened wall cases, respectively^25^.

Fluid and solid mesh was generated in ANSYS ICEM-CFD and ANSYS WORKBENCH-MESH, respectively. The mesh independence was considered achieved when the variation of $TAWSS$, $OSI$, and $RRT$ were less than 3% between two successive simulations (see Supplementary materials). The fluid mesh consisted of 2,474,927 elements with 10 prismatic layers near the wall, and the solid mesh for the arterial wall thickness of 0.53 mm and 0.77 mm contained 61,432 elements and 79,252 elements, respectively (Fig. 1b). The pulsatile transient simulations were carried out for three full cardiac cycles with the uniform time step of 0.01 s. The data for analysis were obtained at the third cardiac cycle.

### Governing equations

It was well known that blood flow is a non-Newtonian fluid in physical, especially for the blood flow in micro-vessels. Many numerical studies on LDL transport problems were based on non-Newtonian assumption by previous researchers^24,26^. Moreover, previous studies suggested that the shear-thinning and viscoelastic of blood are essential factors that affect the blood flow under various flow rates^27,28^. The non-Newtonian models such as sPTT model, Giesekus model, and Carreau model can obtain relatively accurate results than using Newtonian model^27^, however, they are not convenient to study the effect of a specific viscosity on the flow. Hence, to study the effect of different viscosity on the LDL uptake, the blood flow here is assumption as homogeneous and incompressible blood with Newtonian rheology, the fluid dynamics in the artery lumen can be described by the Navier–Stokes equation. The governing equations of mass, momentum, and species were as follows,

1 $$\nabla \cdot \mathbf{u}=0$$

2 $$\rho \left(\frac{\partial \mathbf{u}}{\partial t}+\mathbf{u}\cdot \nabla \mathbf{u}\right)-\upmu {\nabla }^{2}{\text{u}}+\nabla p-{\varvec{F}}=0$$

3 $$\frac{\partial C}{\partial t}+\mathbf{u}\cdot \nabla C-D{\nabla }^{2}C=0$$

where $\mathbf{u}$ and $p$ are the fluid velocity vector and pressure, $\rho$ ($\text{ 1,050 kg}/{\text{m}}^{3}$) and $\mu$ ($0.0035\text{  Pa}\cdot \text{ s}$ and $0.0040\text{  Pa}\cdot \text{ s}$ for normal and hyper-viscosity cases, respectively) are the blood density and viscosity, ${\varvec{F}}$ is the fluid domain body force (in our present study, ${\varvec{F}}$ is zero), $C$ is the *LDL* concentration, and $D$ is the free diffusivity coefficient of *LDL* which could be set as $5.983\cdot {10}^{-12} {\text{m}}^{2}{\text{s}}^{-1}$^29^.

The mechanism of the *LDL* transport into the vessel wall is in large measure determined by the endothelium. In this study, a three-pore model was introduced to describe *LDL* uptake on the left coronary arterial wall (see Supplementary materials), which was taking account of the contribution of the vesicular pathway, normal junctions, and leaky junctions as well as employing the local $WSS$ to obtain the total *LDL* mass flux^11,14,30,31^.

The arterial wall was considered as an elastic homogeneous material with a density of $1075\text{  kg}/{\text{m}}^{3}$, a Poisson’s ratio of $0.45$, and an isotropic Young’s modulus (*E*) of $3\text{  MPa}$^32^. The conservation equation of the solid part can be derived from Newton's second law of motion. The elastic-dynamic equation can be represented by

4 $${\rho }_{s}\ddot{{d}_{s}}-\nabla {\sigma }_{s}-{f}_{s}=0$$

where ${\rho }_{s}$ is the density of arterial wall, $\ddot{{d}_{s}}$ is the acceleration within the solid region,${\sigma }_{s}$ is the Cauchy stress tensor, and ${f}_{s}$ is the solid domain body force.

### Boundary conditions and computation procedures

**Figure 2:**
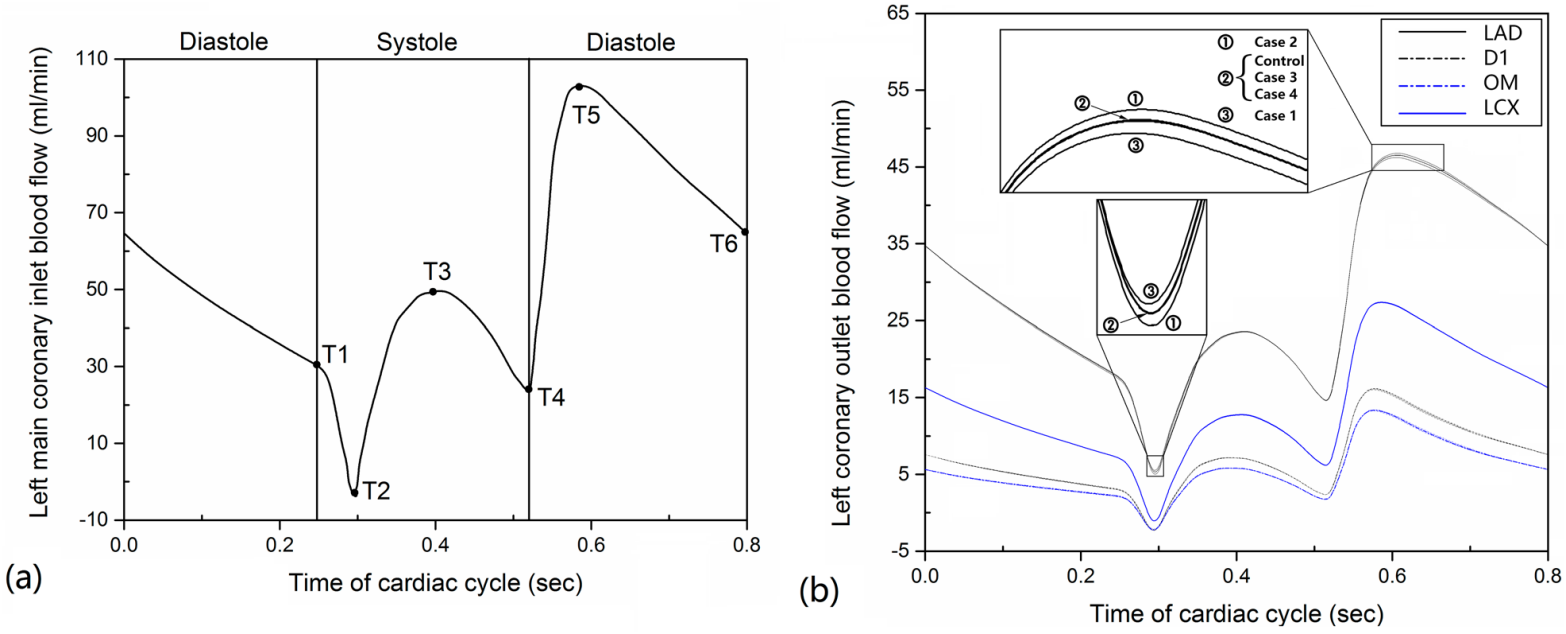
Pulsatile blood flow in human left coronary arteries. (**a**) A pulsatile inlet flow profile of the *LMA*. (**b**) Pulsatile outlet flow profiles of four coronary branch ends for the five simulation cases. Inserts are the pulsatile outlet flow profiles of the *LAD*. Case 1, Case 2, Case 3, and Case 4 respectively represent the condition of rigid wall, blood viscosity, blood pressure, wall thickness. Control represent the healthy condition.

A pulsatile flat inlet flow profile in the left main coronary artery (*LMA*) was set based on a previous study (Fig. 2a)^33^. Pressure-outlet boundary conditions were used at the four inferior ends of the fluid domain, and non-slip boundary condition was specified at the luminal wall. Fully constrained boundary conditions were applied on the edge of the *LMA*, left anterior descending artery (*LAD*), first diagonal branch of the *LAD* (*D1*), left circumflex artery (*LCX*) and left obtuse marginal branch (*OM*) to give stability to the structural calculation. ${\text{P}}_{\text{adv}} = 17.5\text{  mmHg}$ and ${\text{P}}_{\text{adv}} = 30\text{  mmHg}$ were applied at the wall for normal and hypertensive cases, respectively^13,33,34^. A constant *LDL* concentration profile ${\text{C}}_{0}$ equal to $2.86\cdot {10}^{-3}\text{  nmol}/{\text{mm}}^{3}$, corresponding to the physiological *LDL* concentration in whole blood (bulk fluid concentration)^13^, was prescribed at the *LMA* inflow section. At each outflow section of the left coronary artery, $\frac{\partial C}{\partial n}=0$ were applied. At the luminal surface, the boundary conditions of *LDL* lumen-to-wall transfer were referenced to previous studies^6,7,21^.

To study the effect of the different factors on the *LDL* transport via endothelium, five cases were performed. The Control case represented a healthy individual. To study the effect of the elastic property of the arterial wall, a Case with a rigid wall was studied. The numerical simulations were performed with ANSYS 14.0 software. The details of five numerical calculations were as follows.

Control: $$\text{ wall thickness}=0.53\text{  mm}$$, $$\text{ viscosity}=0.0035\text{  Pa}\cdot \text{ s}$$, $$\text{ blood pressures}=70\text{  mmHg}$$

Case 1: $$\text{ rigid wall}$$, $$\text{ viscosity}=0.0035\text{  Pa}\cdot \text{ s}$$, $$\text{ blood pressures}=70\text{  mmHg}$$

Case 2: $$\text{ wall thickness}=0.53\text{  mm}$$, $$\text{ viscosity}=0.0040\text{  Pa}\cdot \text{ s}$$, $$\text{ blood pressures}=70\text{  mmHg}$$

Case 3: $$\text{ wall thickness}=0.53\text{  mm}$$, $$\text{ viscosity}=0.0035\text{  Pa}\cdot \text{ s}$$, $$\text{ blood pressures}=120\text{  mmHg}$$

Case 4: $$\text{ wall thickness}=0.77\text{  mm}$$, $$\text{ viscosity}=0.0035\text{  Pa}\cdot \text{ s}$$, $$\text{ blood pressures}=70\text{  mmHg}.$$

### Hemodynamic parameters

The characteristic time of endothelial cell response to shear stress in terms of mitosis or apoptosis was much longer than that of shear oscillation^35^. Therefore, the following time-averaged hemodynamic parameters were used to characterizing the response of endothelial cells to the blood flow^36^.

5 $$TAWSS=\frac{1}{T}{\int }_{0}^{T}\left|\mathbf{W}\mathbf{S}\mathbf{S}\right|\cdot dt$$

6 $$OSI=0.5\left[1-\left(\frac{\left|{\int }_{0}^{T}\mathbf{W}\mathbf{S}\mathbf{S}\cdot dt\right|}{{\int }_{0}^{T}\left|\mathbf{W}\mathbf{S}\mathbf{S}\right|\cdot dt}\right)\right]$$

7 $$RRT=\frac{1}{TAWSS\cdot \left(1-2\cdot OSI\right)}$$

8 $$TAWSSG=\frac{1}{T}{\int }_{0}^{T}\sqrt{{\left(\left|\frac{\partial \mathbf{W}\mathbf{S}\mathbf{S}}{\partial x}\right|\right)}^{2}+{\left(\left|\frac{\partial \mathbf{W}\mathbf{S}\mathbf{S}}{\partial y}\right|\right)}^{2}+{\left(\left|\frac{\partial \mathbf{W}\mathbf{S}\mathbf{S}}{\partial z}\right|\right)}^{2}}\cdot dt$$

where *T* is the duration of the cardiac cycle. $\mathbf{W}\mathbf{S}\mathbf{S}$ is the *WSS* vector which is defined as the scalar dot product of the unit normal vector to a surface and the viscous stress tensor^37^. $\partial /\partial x$, $\partial /\partial y$, and $\partial /\partial z$, are the partial derivatives with respect to the $x$, $y$ and $z$ coordinates, respectively. $TAWSS$ represented the time-averaged magnitude of the *WSS* during the cardiac cycle(s). If $TAWSS$ is less than 1 Pa, intimal thickening occurs^38^. The *OSI* is employed to describe the oscillatory nature of the *WSS* throughout the cardiac cycle; and the *RRT* is included the effects *OSI* and $TAWSS$, that is employed to identify regions in which high particle residence time occur^37,38^.

### Ethics approval

This study was approved by the Research Ethics Committee of the Guangdong General Hospital, Guangdong Academy of Medical Sciences, and was performed per the Declaration of Helsinki.

### Informed consent

The need of informed consent was waived by the Research Ethics Committee of the Guangdong General Hospital, Guangdong Academy of Medical Sciences.

## Results

### The change of blood flow flux with the cardiac cycle

For all the cases, *LAD* has the largest flow flux over all the time, followed by *LCX*, *D1,* and *OM*, respectively (Fig. 2b). Moreover, there is a deviation of flow rate among the five cases during the cardiac cycles, especially at the periods of early-systole and early-diastole. This suggested that the blood flow flux at the periods of early-systole and early-diastole are easily disturbed by external conditions. In detail, at the period of early-systole, Case 1 shows the highest blood flow rate in the arterial ends of LAD while Case 2 indicates the smallest flow rate (see “insert views” in Fig. 2b). The flow rate among Control and Case 3 and Case 4 have no significant difference. However, at the period of early-diastole, the situations of flow rate are totally reversed (see “insert views” in Fig. 2b), at which Case 2 shows the highest blood flow rate.

### The streamline profiles at the different periods during the cardiac cycle

**Figure 3:**
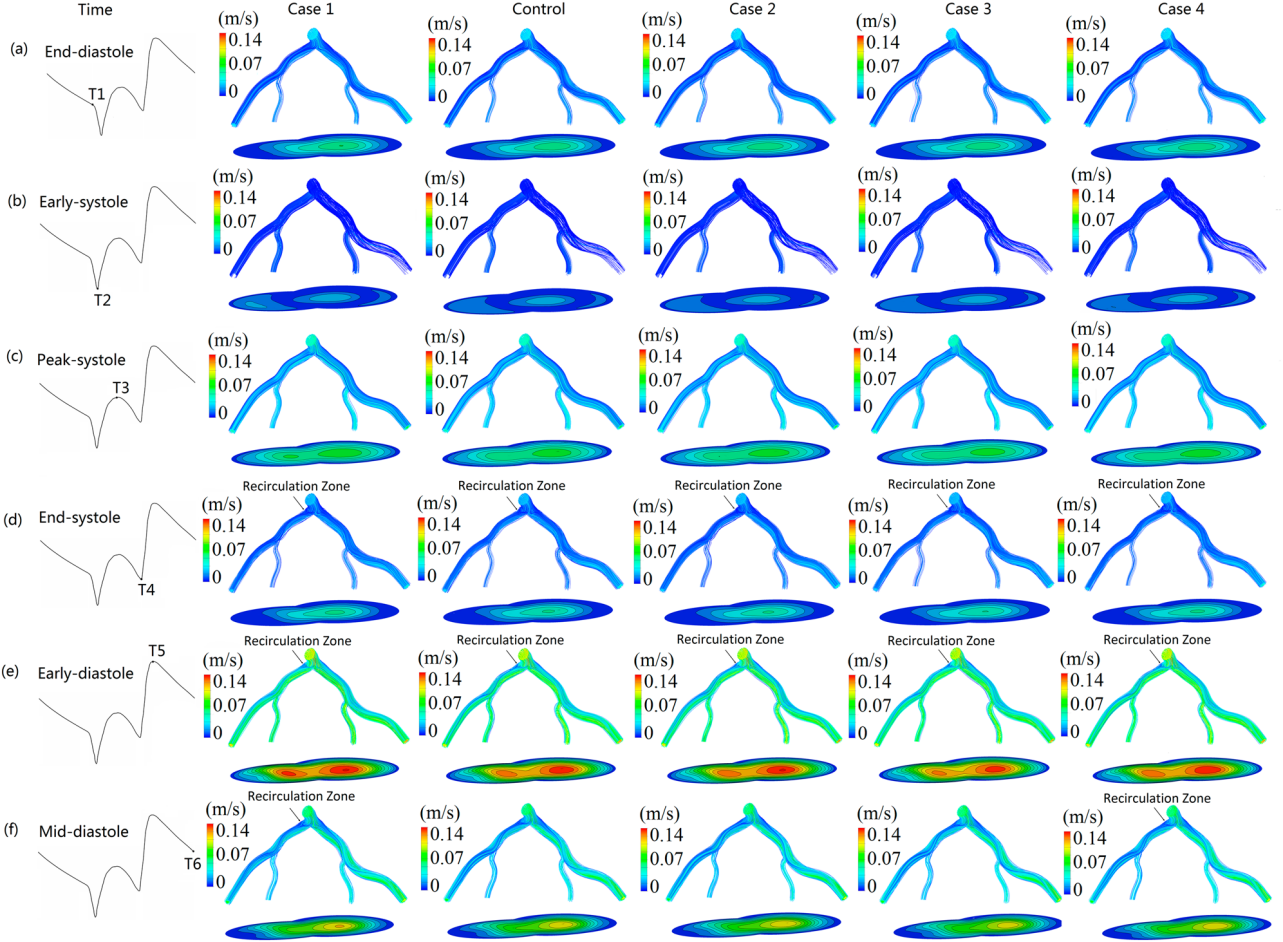
Streamlines and velocity profiles during the cardiac cycle for the five simulation cases. The marked points T1–T6 (**a**–**f**), respectively denote end-diastole, early-systole, peak-systole, end-systole, early-diastole, mid-diastole.

Figure 3 illustrates velocity profiles during the cardiac cycle for the five simulations. The flow patterns at the six different periods are significant difference from each other. Precisely, the coronary system keeps the smallest velocity at the period of early-systole while it holds the largest velocity at the period of early-diastole. At the period of early-diastole, strong secondary flow and recirculation zone at bifurcation regions can be observed. At the period of mid-diastole, the size of recirculation zone at bifurcation regions are different among the five cases (Fig. 3f). In detail, Case 1 has the largest recirculation zone in the bifurcation regions, followed by Case 4. However, there is no clear recirculation zone appearing in the bifurcation regions for the simulations of Case 2, Case 3, and Control. This suggested that rigid and thickened arterial hold the most complex flow at the bifurcation regions. As the elasticity of vessel decreased when the arterial wall thickness increased^39^, consequently, the thickened wall of the coronary artery will have lower elastic property when compared with non-thickened ones. In other words, superior elasticity of the arterial wall could decrease the complexity of the local flow field in the coronary bifurcation system.

### The distribution of hemodynamic parameters on the luminal wall

**Figure 4:**
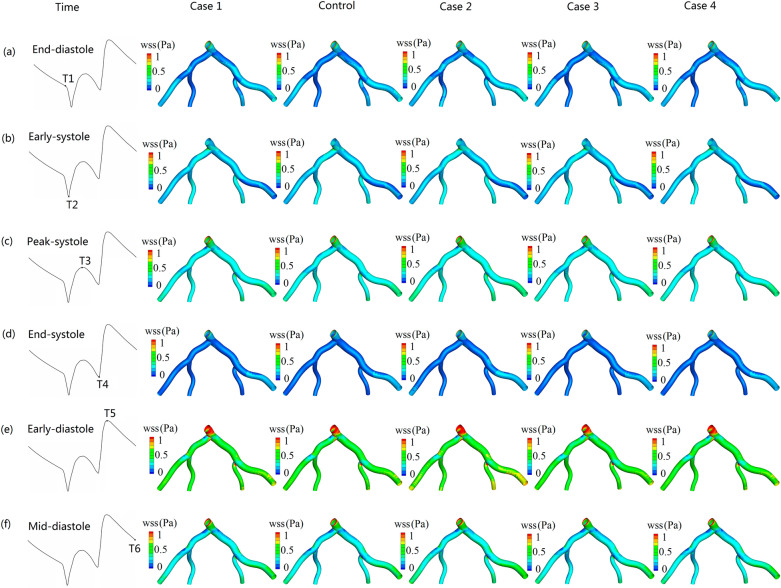
Wall shear stress distributions during the cardiac cycle for the five simulations. (**a**–**f**), respectively, represent the period of end-diastole, early-systole, peak-systole, end-systole, early-diastole, mid-diastole.

Figure 4 shows the $WSS$ distributions during the cardiac cycle for the five simulations. The results show that the $WSS$ at the deceleration period of cardiac cycle is lower than that at the acceleration period of cardiac cycle, especially at the period of end-deceleration. For all the simulations, at the period of end-systole, the luminal surface has the lowest $WSS$, followed by the period of end-diastole. However, at the period of early-diastole, the luminal surface has the highest $WSS$.

**Figure 5:**
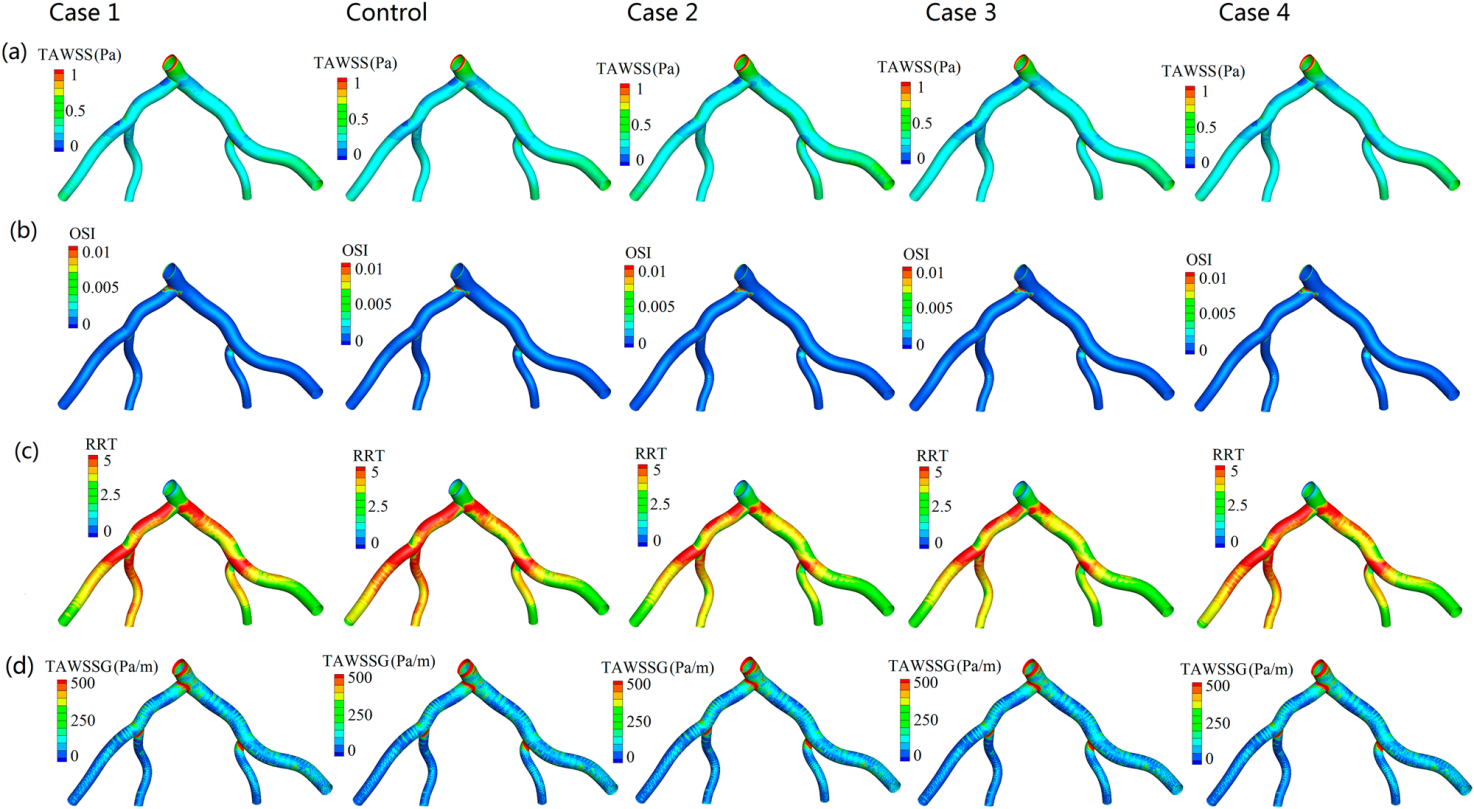
Wall shear stress-based hemodynamic indicators distributions for the five simulations. (**a**) The distribution of time-averaged wall shear stress. (**b**) The distribution of oscillatory shear index. (**c**) The distribution of relative residence time. (**d**) The distribution of time-averaged wall shear stress gradient. $TAWSS$ time-averaged wall shear stress, $OSI$ oscillatory shear index, $RRT$ relative residence time, $TAWSSG$ time-averaged wall shear stress gradient.

Figure 5a–d respectively displays the distribution of time-averaged hemodynamic parameters of $TAWSS$, $OSI$, $RRT$ and $TAWSSG$ for the five cases. The distribution of these time-averaged hemodynamic parameters for all the simulation conditions are similar among the five cases. The $TAWSS$ of Case 2 is slightly higher than that of the other four cases (Fig. 5a). The regions with low $TAWSS$, high $OSI$ are near the vascular bifurcations (Fig. 5a,b). Moreover, the $TAWSS$ in upstream and outside of *LCX* is relatively lower when it is compared with the downstream and inner wall. Results show that high-$RRT$ regions on the arterial wall are mainly located at the low-$TAWSS$ regions (Fig. 5a,c). Large areas of low $TAWSSG$ are located at the downstream of four branch arteries (Fig. 5d). Extremely high $TAWSSG$ can be easily found in the regions of vascular bifurcation, such as the bifurcation of *LAD* and *LCX*.

### The distribution of three-pore model related indicators on the luminal wall

**Figure 6:**
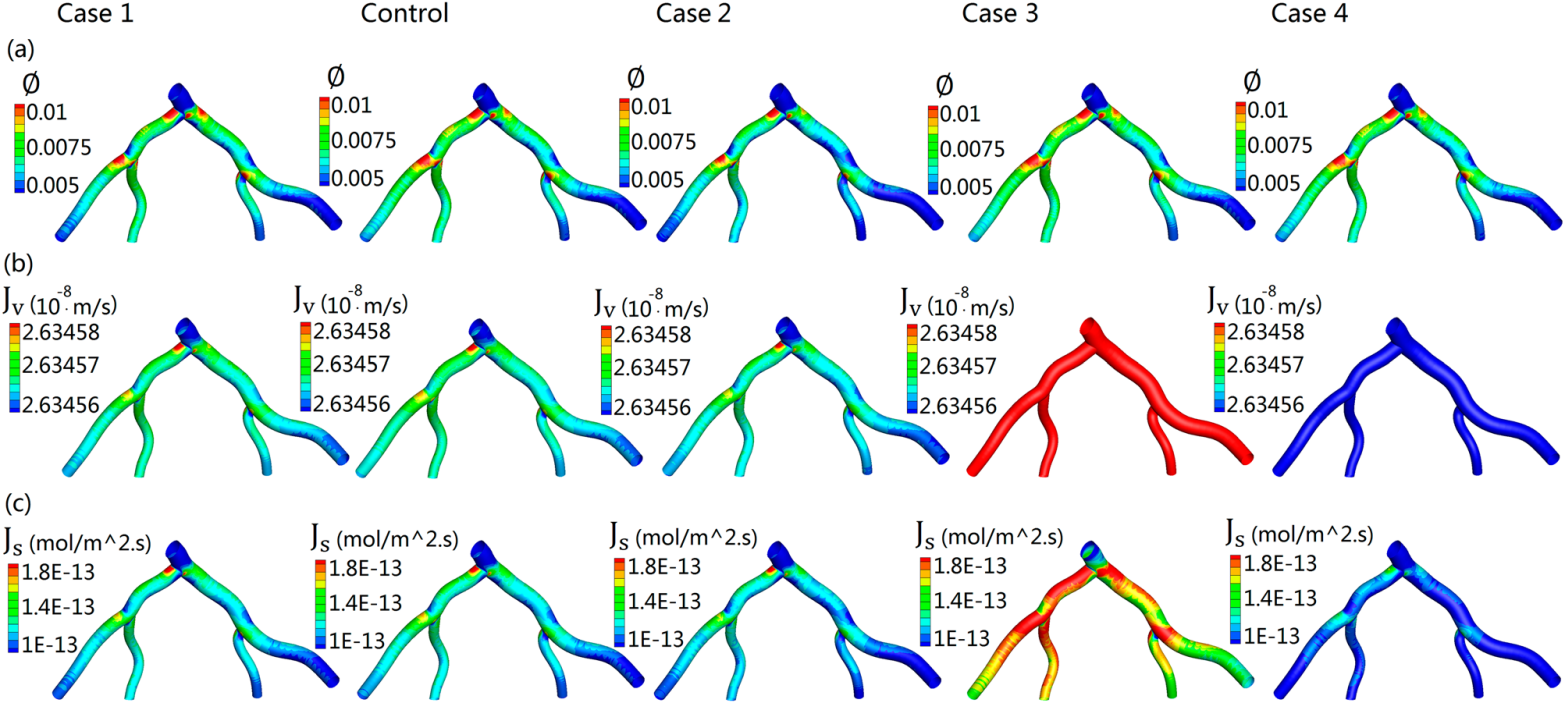
Three-pore model related indicators for the five simulations. (**a**) The distribution of the fraction of leaky junctions on endothelium (${ \varnothing }$) of human left coronary arteries. (**b**) The distribution of total plasma volume flux (${\text{J}}_{\text{v}}$) through the endothelium. (**c**) The distribution of total mass flux of *LDL* (${\text{J}}_{\text{s}}$).

Figure 6a showes the fraction of leaky junctions (${ \varnothing }$) distribution on endothelium for the five simulation cases. The distribution of ${ \varnothing }$ on the arterial wall is similar among the five cases. The regions of high ${ \varnothing }$ consistent with the places of low $TAWSS$ regions (Figs. 5a, 6a). Figure 6b,c respectively shows the fluid volume fluxes and *LDL* mass fluxes through the endothelium for the five simulation cases. Higher fluid volume flux (${\text{J}}_{\text{v}}$) regions are mainly located at the outer wall of *LAD* and *LCX* where the $TAWSS$ was relatively low (Figs. 5a, 6b). The values of ${\text{J}}_{\text{v}}$ for Case 3 are significantly higher than that of the other four cases, while Case 4 had the lowest ${\text{J}}_{\text{v}}$ among the five cases (Fig. 6b). Moreover, the value of ${\text{J}}_{\text{v}}$ on the *LCX* and *OM* is relatively higher than that on the *LAD* and *D1*. The highest value occurs at bifurcations where the LMA stem branches into *LAD* and *LCX*, the value at which is as high as $2.63458\times {10}^{-8} \text{m}/\text{s}$. Low ${\text{J}}_{\text{v}}$ regions are mainly located at inner side of twisting arteries, the lowest value is under $2.63456\times {10}^{-8}\text{m}/\text{s}$. According to the three pore-model, the value of ${\text{J}}_{\text{s}}$ into the arterial wall is not only determined by volume fluxes, but also by the concentration of LDL on the near wall. Moreover, the LDL transport in the bloodstream is calculated by the convection–diffusion equation (Eq. 3). As the distribution of all the hemodynamic parameters (TAWSS, OSI, RRT, TAWWSG) for the five different cases is similar from each other (Fig. 5a–d), hence, the distribution of LDL on the luminal wall would be also similar too. Nevertheless, LDL concentration profiles would be changed very much at the different places in the same model of the arterial wall surfaces. Figure 6c show the distribution of *LDL* mass flux (${\text{J}}_{\text{s}}$) through the endothelium. Case 3 has the highest ${\text{J}}_{\text{s}}$ while Case 4 has the lowest ${\text{J}}_{\text{s}}$ among the five cases. The regions with high value of ${\text{J}}_{\text{s}}$ are always occurred at *LCX* and low value regions are mainly located at the downstream of *LAD* and *LCX* and the upstream of *LMA*. The highest value of ${\text{J}}_{\text{s}}$ are mainly located at the recirculation zones where the $WSS$ is usually very low. Overall, the distribution trend of ${\text{J}}_{\text{s}}$ is similar with ${\text{J}}_{\text{v}}$, but the absolute values are not completely the same between them. In addition, although the color-coded maps of ${ \varnothing }$ didn't appear to show much difference among the five different cases, ${\text{J}}_{\text{v}}$ and ${\text{J}}_{\text{s}}$ are still showing significant differences among the five different cases, especially case 3 (increased blood pressure) and case 4 (increased wall thickness).

### The distribution of the percentage difference of the ${J}_{v}$ and ${J}_{s}$ on the luminal wall

**Figure 7:**
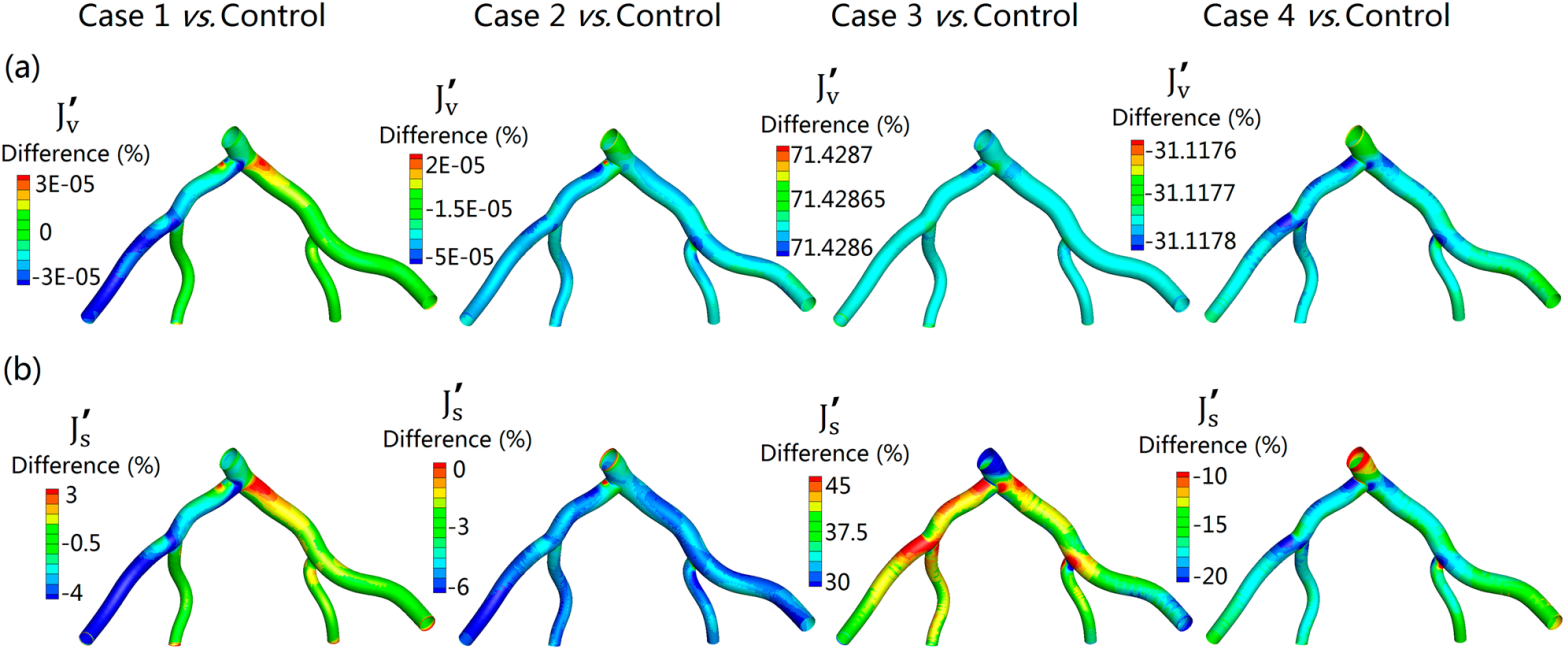
Comparison of the three-pore model related indicators for the five simulations. (**a**,**b**) respectively represent the percentage difference of ${\text{J}}_{\text{v}}$ (${\text{J}}_{\text{v}}^{{ {\prime}}}$) and ${\text{J}}_{\text{s}}$ (${\text{J}}_{\text{s}}^{{ {\prime}}}$) between different experimental groups and Control simulation. (**a**) ${\text{J}}_{\text{v}}^{{ {\prime}}}=\left({{\text{J}}_{\text{v}}}_{\text{Experimental groups}}-{{\text{J}}_{\text{v}}}_{Control}\right)/{{\text{J}}_{\text{v}}}_{Control}$, (**b**) ${\text{J}}_{\text{s}}^{{ {\prime}}}=\left({{\text{J}}_{\text{s}}}_{E\text{ xperimental groups}}-{{\text{J}}_{\text{s}}}_{Control}\right)/{{\text{J}}_{\text{s}}}_{Control}$. Where Case 1 *vs.* Control: the distribution of the percentage difference between Case 1 with rigid wall and Control. Case 2 vs. Control: the distribution of the percentage difference between Case 2 with increased blood viscosity and Control. Case 3 vs. Control: the distribution of the percentage difference between Case 3 with hypertension and Control. Case 4 vs. Control: the distribution of the percentage difference between Case 4 with thickened wall and Control.

Figure 7a,b respectively shows the percentage difference of ${\text{J}}_{\text{v}}$ (${\text{J}}_{\text{v}}^{{{\prime}}}$) and ${\text{J}}_{\text{s}}$ (${\text{J}}_{\text{s}}^{{ {\prime}}}$) between Case (1, 2, 3, 4) and Control case. The relatively high values of ${\text{J}}_{\text{v}}^{{ {\prime}}}$ between rigid and elastic models (Case 1 vs. Control) are located at the regions of the outer wall of the bifurcation (from LMA into LAD and LCX) (slightly higher than 0.00003%). Moreover, all the ${\text{J}}_{\text{v}}^{{{\prime}}}$ of Case 1 vs. Control and Case 2 vs. Control in most regions are significant small (less than 0.00005%). Therefore, the value of ${\text{J}}_{\text{v}}^{{{\prime}}}$ between Case 1 and Case 2 and Control can be neglected. However, ${\text{J}}_{\text{v}}^{{{\prime}}}$ between Case 3 and Control is over 70% and the average value of ${\text{J}}_{\text{v}}^{{{\prime}}}$ between Case 4 and Control is over 30% (Fig. 7a). This phenomenon is similar to the value of ${\text{J}}_{\text{s}}^{{ {\prime}}}$, although the values between them are not completely the same (Fig. 7b). This suggested hypertension increased the plasma fluid and *LDL* mass flux through the endothelium, while the thickened arterial wall decreases this process.

## Discussion

This paper applied an $FSI$ method to study the influence of different physical factors on the *LDL* uptake from flowing blood to the arterial wall in the left coronary artery. The results revealed that: (1) High elastic property of the arterial wall can decrease the complexity of the local flow field in the coronary bifurcation system. (2) The regions of high *LDL* uptake are located in the regions of low wall shear stress. (3) Hypertension can promote *LDL* uptake in the arterial wall, quite the contrary, thickened arterial wall can decrease the uptake of *LDL*.

The important feature of atherosclerosis is the narrowing of the arteries. It can block the blood flowing in the vessel. Atherosclerosis is characterized by patchy intimal plaques that encroach on the lumen of medium-sized and large arteries; the plaques contain lipids, inflammatory cells, smooth muscle cells, and connective tissue. As we know, risk factors of atherosclerosis include dyslipidemia, LDL, diabetes, cigarette smoking, family history, sedentary lifestyle, obesity, and hypertension^40^. There was much evidence suggesting that the initiation and progression of the atherosclerotic disease involved a significant accumulation of LDL in the arterial wall^5^. Moreover, a large number of LDL uptake is the key factor leading to the accumulation of LDL. Therefore, early accumulation of LDL could be the most important reason for the intimal thickening, which is the first step of the arterial narrowing (initiation of intimal thickening). Although our present study did not directly predict intimal thickening, it still can be effective in predicting the distribution and uptakes of LDL in the arterial wall. Hence, the present results may partially predict the intimal thickening according to the amounts of LDL uptakes in the coronary artery.

Previous studies showed that vascular geometric structure, such as the branches, curvatures, and bifurcations have a significant effect on the local flow field distribution^41–44^. Our recent research also confirms that complex flow, such as strong secondary flow and recirculation flow, are easily discovered around the bifurcation regions. In addition, the blood flow at the wide-angle bifurcations, such as *LMA* stem branches into *LAD* and *LCX*, are more complex when compared with the narrow-angle bifurcations (e.g. *LAD* branches into the *D1* and the *LCX* branches into the *OM*) (Figs. 3, 4, 5). Our present study further indicates that in addition to the influence of vascular geometric structure on the blood flow field, other factors, such as the pulsatile and the elasticity of the arterial wall, also have much effect on the blood flow field (Figs. 2, 3, 4). It is shown that the flow patterns at different periods during the cardiac cycles are significantly different. The results also show that rigid wall condition leads to the largest recirculation zone and the most complex streamlines at the bifurcation regions when compared to the condition of the vascular wall with elastic properties (Fig. 3). Moreover, when flowing blood flows in the wall-thickened coronary artery, the flow field is also complex than that of the un-thickened ones. This indicates that the effect of the rigid wall and the thickened wall on the flow field are very similar. Study by Bastida et al. showed that thickened arterial wall has lower elastic property when compared with thinner arterial wall^39^. This suggested that the higher elasticity of arterial wall can decrease the complexity of the local flow profiles. The dysfunction of the endothelium led to atherogenesis, this process is stimulated by the complex hemodynamic forces^12^. Therefore, the elasticity of human blood vessels may play a self-protection role in preventing vascular atherosclerotic lesions.

For all the simulations, the regions of high blood plasma flux coincide with the regions of high *LDL* flux and the regions of low $TAWS\text{ S}$, high $OSI$, high $RRT$, and high $TAWSSG$ (Figs. 5, 6). Other previous studies also indicated that LDL accumulation increased with lower wall shear stress^26,45^. Studies by John et al. suggested that the transport processes of *LDL* are determined by both blood flow and endothelium^46^. It was showed that the fluid mechanics may be influential when mass transfer coefficient is much smaller than the surface reaction rate coefficient (the process was termed “fluid-phase-limited”), conversely, luminal wall could be influential when mass transfer coefficient is greater than the surface reaction rate coefficient (the process was termed “wall-limited”)^46^. Previous studies show that blood plasma flux can not only through leaky junctions, but also through normal junctions^46,47^. However, *LDL* particles are too large to pass through normal junctions^11^, which means that the effect of convection effect of blood plasma transmural flow on *LDL* accumulation are largely affected by the percentage of leaky junctions, not normal junctions. There are two main ways for *LDL* particles transport through the endothelial wall, the portion of *LDL* transport via vesicular transcytosis mechanism is less than 10%, whereas the portion of *LDL* transport through leaky junctions mechanism is over 90%^35^. This suggested that the *LDL* transport into the arterial wall is largely affected by the number of leaky junctions. According to the three-pore model, the fraction of leaky junctions on endothelium is a function of local $WS\text{ S}$^11^. The regions of low $WS\text{ S}$ consistent with the places of a higher number of leaky junctions (Figs. 4, 5a, 6a). Hence, in the low $WS\text{ S}$ regions, the transmural resistance on the endothelium to flow is decreased considerably^33^. This leads to an increase in the flux of blood plasma in those low $WS\text{ S}$ regions of the coronary wall. Due to the convection effect of blood plasma in the leaky junctions, the increased plasma flux, in turn, leads to increased transportation of *LDL* particles from the lumen into the arterial wall (Fig. 6b,c).

On the other hand, increased transmural pressure also leads to an increased filtration velocity through leaky junctions, consequently, resulting in an increased convective flux of *LDL* through the endothelium. These results are consistent with previous numerical findings^13,26,48^. There are many reasons for this phenomenon. Firstly, according to the principle of fluid dynamics, higher blood pressure is usually accompanied by lower flow velocity, hence leads to lower wall shear stress. The effect of low wall shear stress on the LDL uptake has been demonstrated above. On the other hand, studies suggested that the increased transmural pressure could induce arterial wall distension and stretch, which leads to an increased number of mitosis and apoptosis^49^. This phenomenon will result in an increased number of leaky junctions, therefore, leads to an increase of endothelial diffusive permeability^50,51^. Moreover, pressure-driven convective flow at higher transmural pressure also enhances *LDL* uptake^13^. It is well accepted that the early atherogenesis tends to be hallmarked by an abnormally high accumulation of *LDL*^7,13^. Our results confirm that plasma flux and *LDL* mass flux of hypertension case is significantly higher than that of the control individual (Fig. 7). This suggested that that hypertension may increase the susceptibility to atherosclerosis by increasing the accumulation of *LDL* on the endothelium. Furthermore, as the blood pressure in the proximal end of coronary is usually greater than that in the distal end, therefore, the uptake of *LDL* is relatively high in the proximal end (Fig. 6). This gives us a hypothesis that the near heart arteries could be more susceptible to atherosclerosis. Therefore, it may partially be explained why atherosclerosis easily happened in large arteries, such as LMA and aorta.

Unlike the effect of blood pressure, the wall thickness played an opposite role on the uptake of *LDL* in coronary. The results in our recent studies show that thickened wall can act to inhibit *LDL* entry into the arterial wall. This may indicate that the earlier formation rate of atherosclerotic plaque is relatively faster. As the atherosclerotic plaque grew, and over time, the growth rate will become slower in the later period of plaque formation. This finding is consistent with the study by Liu et al. that the atherosclerotic plaque grows at a decreasing rate in the progression process^52^. Moreover, although high blood viscosity condition increases the $TAWSS$ on the arterial surface, it played a minor role in the effect of *LDL* uptake (Figs. 5a, 6c). This may because blood viscosity may affect the LDL transport only in the vessel lumen, but the uptake of LDL in the arterial wall could be mainly dominated by the surface passages of the endothelium^53^.

*Limitations:* All the simulations of the present study are based on a flat inflow boundary condition, and the ends of the artery branches are prescribed as constant pressures. This may not perfectly reproduce the pulse wave propagation. However, it should be pointed out that our method for the boundary conditions setting are still reasonable. It can help us to predict reasonable results on the distribution trend because the present boundary conditions set could only affect the absolute values, but not the trend. Furthermore, the arterial wall in our present study is regarded as a sing-layer with a constant thickness. The distribution of vascular wall thickness may be different along the luminal wall of the coronary arteries under the physiological conditions. However, it is unrealistic for us to build a model with different wall thicknesses along the vessel because the patient-specific CTA image used for generating the computational models is from a patient by medical examination in the hospital. There is no accurate information about arterial wall thickness, we can only obtain the contour of the blood vessel wall from the CTA image. Therefore, in this study, the parameter data of wall thickness are from the previously published paper. Another limitation of the present study is that all the simulations are based on a Newtonian fluid assumption with a constant viscosity. The shear-thinning and viscoelastic properties of blood can affect the blood flow under various flow rates. Nevertheless, this study can be convenient for us to quantitatively study the effect of different viscosities on the LDL accumulation.

## Conclusions

By using the numerical method of $FSI$ technique for studying the *LDL* uptake in human left coronary arteries, we can know that high elastic property of the arterial wall can decrease the complexity of the local flow field in the coronary bifurcation system. This may suggest that the elastic property of the arterial wall is good for preventing atherosclerotic disease. Moreover, we can also know that the places of high levels of *LDL* uptake coincided with the regions of low wall shear stress; the thickened arterial wall can decrease the uptake of *LDL*; the present study revealed that hypertension significantly promoted LDL accumulating in the arterial wall. This study found that hypertension may play the chief culprit role in atherosclerotic lesions. More importantly, as the places of atherosclerosis lesion usually consistent with the regions with high LDL uptake, hence the present computer strategy has a great potential for the prediction of the local atherosclerosis lesion.

## Supplementary Information

Supplementary Information

## Data Availability

The datasets supporting the conclusions of this article were included within the main paper.
