# Supplementary material for: Fluid–structure interactions (FSI) based study of low-density lipoproteins (LDL) uptake in the left coronary artery
Source: Sci Rep. 2021 Feb 26;11:4803. doi: 10.1038/s41598-021-84155-3 (PMC7910311; doi:10.1038/s41598-021-84155-3)
Supplement: Supplementary file 1 — Supplementary Information [file 41598_2021_84155_MOESM1_ESM.docx]

**Supplementary materials of fluid-structure interactions (FSI) based study of low-density lipoproteins (LDL) uptake in the left coronary artery**

Xueping Chen^1^, Jian Zhuang^2^, Huanlei Huang^2^, Yueheng Wu^1,2*^

^1^Institute of Biomechanics, School of Bioscience and Bioengineering, South China University of Technology, Guangzhou, 510006, P.R. China;

^2^Department of Cardiovascular Surgery, Guangdong Cardiovascular Institute, Guangdong Provincial Key Laboratory of South China Structural Heart Disease, Guangdong Provincial People's Hospital & Guangdong Academy of Medical Sciences, School of Medicine, South China University of Technology, Guangzhou, 510080, P.R. China;

Corresponding author: E-mail address: wuyueheng@gdph.org.cn (Y. Wu), Tel.: +86 020 83827812(51153),

**1.** **Methods**

*1.1 Determination of volume (solutions of* *blood plasma) fluxes and mass (solutes of LDL)* *fluxes via the endothelium*

The mechanism of the *LDL* transport in the vessel wall is in large measure determined by the endothelium. This layer causes the highest hydraulic and mass transfer resistance across the arterial wall due to its small pore size. Therefore, any factors that cause a change in the valid pore size have a significant impact on the flow and mass transfer behavior within the endothelium layer, and thus within the entire wall. In our present study, a three-pore model was introduced to describe blood plasma and *LDL* transport through the left coronary artery endothelium, which is taking account of the contribution of the vesicular pathway, normal junctions, and leaky junctions as well as employing the local $WSS$ to obtain the total volume and mass flux ^1-4^. Before well describing this three-pore model, we firstly calculate the fraction of leaky junctions on endothelium,$\emptyset$, which is defined as the ratio of the area leaky cells to the area of all cells, where $\emptyset$ is a function of local $WSS$.

| $\mathrm{SI}=0.380e^{-0.790WSS}+0.225e^{-0.043WSS}$ | | (1) |
| --- | --- | --- |
| $⋕MC=0.003797e^{14SI}$ | | (2) |
| $⋕LC=0.307+0.805\cdot\left( ⋕MC \right)$ | | (3) |
| $\emptyset=\frac{⋕LC\times\pi R_{\mathrm{Cell}}^{2}}{S_{\mathrm{unit}}}$ | (4) | |

Where $\mathrm{SI}$ is the endothelial cells shape index, $⋕MC$ and $⋕LC$ are the number of mitotic cells and leaky cells, respectively, at the regions with specific shape index.$R_{\mathrm{Cell}}$ is the radius of a single endothelial cell taken as 15 $\mu m$ ^5^, and $S_{\mathrm{unit}}$ is a periodic circular unit area, set to be 0.64 $\mathrm{mm}^{2}$ ^6^. $WSS$ (in Pa) is the intraluminal wall shear stress over the endothelium obtained from fluid-structure interactions simulations.

In the three-pore theorem, the total volume flux $J_{v}$ and the total mass flux $J_{s}$ can be achieved by summing the flux over each single pathway, the relationships are

| $J_{v}=J_{v,v}+J_{v,nj}+J_{v,lj}$ | (5) |
| --- | --- |
| $J_{s}=P_{\mathrm{app}}C_{\mathrm{end}}$ | (6) |
| $P_{\mathrm{app}}=P_{v}+P_{app,nj}+P_{app,lj}$ | (7) |

Where $J_{v,v}$, $J_{v,nj}$, $J_{v,lj}$ are the volume flux through the vesicular pathway, normal junctions, and leaky junctions, respectively. $P_{\mathrm{app}}$ is the total apparent permeability of the endothelium, and $C_{\mathrm{end}}$ is the lumen side concentration of *LDL* at the endothelium, which is obtained from numerical simulations. $P_{v}$ is the permeability of the vesicular pathway, $P_{app,nj}$ and $P_{app,lj}$ are the apparent permeability of the normal junctions and the leaky junctions, respectively ^1^.

Previous studies showed that blood plasma flux occurs only through normal junctions and leaky junctions, and it can only carry solutes with a radius smaller than 2nm when filtering through the normal junctions, this pore size is smaller than the radius of *LDL* molecule ($a= 11 \mathrm{nm}$) ^7^, therefore, we can obtain that $J_{v,v}=0$, $P_{app,nj}=0$. Previous researches also showed that the percentage of *LDL* passage via vesicular pathway is less than 10% ($P_{v}=1.92\times{10}^{-11}m/s$), in contrast to more than 90% of *LDL* particles transport into the artery wall via leaky junctions ^8-10^. The apparent permeability of the leaky junctions $P_{app,lj}$ can be expressed by

| $P_{app,lj}=P_{\mathrm{lj}}Z_{\mathrm{lj}}+J_{v,lj}\left( 1-\sigma_{f,lj} \right)$ | (8) |
| --- | --- |

Where $P_{\mathrm{lj}}$ and $\sigma_{f,lj}$ are the diffusive permeability and the solvent-drag reflection coefficient of the leaky junctions, respectively.$Z_{\mathrm{lj}}$ is the fractional reduction factor in solute concentration gradient at the entrance of leaky junction pore.$P_{\mathrm{lj}}$, $\sigma_{f,lj}$ and $Z_{\mathrm{lj}}$can be given as ^1,3,4,11^

| $P_{\mathrm{lj}}=\frac{w^{2}}{3}\frac{4w\emptyset}{R_{\mathrm{Cell}}}$ | (9) |
| --- | --- |
| $\sigma_{f,lj}=1-\left( 1-\frac{3}{2}\alpha_{\mathrm{lj}}^{2}+\frac{1}{2}\alpha_{\mathrm{lj}}^{3} \right)\left( 1-\frac{1}{3}\alpha_{\mathrm{lj}}^{2} \right)$ | (10) |
| $Z_{\mathrm{lj}}=\mathrm{Pe}_{\mathrm{lj}}/\left( e^{\mathrm{Pe}_{\mathrm{lj}}}-1 \right)$ | (11) |

Where $w$ is the half-width of leaky junctions equal to 20 $\mathrm{nm}$, $R_{\mathrm{Cell}}$ is the radius of a single endothelial cell, $\alpha_{\mathrm{lj}}=a/w$ is the ratio of the radius of *LDL* to the half-width of the leaky junctions, $\emptyset$ is the ratio of the area of leaky cells to the area of all cells on the endothelium, $\mathrm{Pe}_{\mathrm{lj}}$ is the modified Péclet number that is defined as the ratio of convective to diffusive fluxes through the leaky junctions, which can be expressed by

| $\mathrm{Pe}_{\mathrm{lj}}=\left[ J_{v,lj}\left( 1-\sigma_{f,lj} \right) \right]/P_{\mathrm{lj}}$ | (12) |
| --- | --- |

The blood plasma trough the endothelium and into the arterial wall was modelled using an electrical analogy in which the flow is driven by a pressure difference and the resistance to the flow entering the arterial wall is given by the endothelial layer, the model can be expressed as ^1^

| $J_{v}=\frac{p_{end}-p_{adv}}{R_{T}}$ | (13) |
| --- | --- |
| $J_{v,lj}=J_{v}\frac{R_{\mathrm{end}}}{R_{\mathrm{lj}}}$ | (14) |
| $J_{v,nj}=J_{v}\frac{R_{\mathrm{end}}}{R_{\mathrm{nj}}}$ | (15) |
| $\frac{1}{R_{\mathrm{end}}}=\frac{1}{R_{\mathrm{nj}}}+\frac{1}{R_{\mathrm{lj}}}=L_{p,nj}+L_{p,lj}$ | (16) |

Where $p_{end}$ is the intraluminal pressures over the endothelium, $p_{adv}$ is the pressures in the media-adventitia interface, $R_{T}=R_{\mathrm{end}}+R_{\mathrm{wall}}$ is the total flow resistance, which formed by the endothelial resistance,$R_{\mathrm{end}}$, and the wall resistance,$R_{\mathrm{wall}}=\frac{\mu_{p}\mathrm{WTH}}{K_{D}}$ ^1,12^ (where $\mu_{p}=0.001Pa\cdot s$,$\mathrm{WTH}$, and $K_{D}=2.0\cdot{10}^{-18}m^{2}$ are the viscosity of blood plasma, thickness of the arterial wall, and the Darcy’s permeability of the arterial wall). $R_{\mathrm{nj}}=1/{L_{p,nj}}$ and $R_{\mathrm{lj}}=1/{L_{p,lj}}$ are the flow resistance through normal junctions and leaky junctions, in which $L_{p,nj}=1.58\cdot{10}^{-9}m/(s\cdot mm\cdot Hg)$ ^13^ and $L_{p,lj}=\frac{w^{2}}{3\mu_{p}l_{\mathrm{lj}}}\frac{4w\emptyset}{R_{\mathrm{Cell}}}$ ^1,14^ are the hydraulic conductivity via normal junctions and leaky junctions, respectively. (where $w$, $\emptyset$, $l_{\mathrm{lj}}=2\mu m$, and $R_{\mathrm{Cell}}$ are the half-width of leaky junctions, the local fraction of leaky junctions, the length of the leaky junction, and the radius of a single endothelial cell.)

*1.2 Mesh independence study*

In this study, the parameters of $TAWSS$, $OSI$, and $RRT$ were used to determine the mesh independence. Mesh independence was considered to be achieved when the values difference between two successive simulations was less than 3%. The mesh independence study was studied based on the Control model: $wall thickness=0.53 mm$, $viscosity=0.0035 Pa\cdot s$, $blood pressures=70 mmHg$. When the mesh independence of this model was reached, the fluid mesh of which will be used for all the other Cases. Moreover, the mesh size parameters of $wall thickness=0.77 mm$ were the same as the $wall thickness=0.53 mm$. In this study, two types of mesh size were compared, for the coarser size mesh (MESH1), the fluid mesh consists of 2,474,927 elements, and the solid mesh for the arterial wall contain 61,432 elements. However, for the denser size mesh (MESH2), the fluid mesh consists of 3,714,390 elements, and the solid mesh for the arterial wall contain 92,270 elements. Figure S1 shows the comparison of $TAWSS$, $OSI$, and $RRT$ between MESH1 and MESH2. The results show that the variation (absolute value of percentage difference) of $TAWSS$, $OSI$, and $RRT$ are all less than 3% between MESH1 and MESH2.


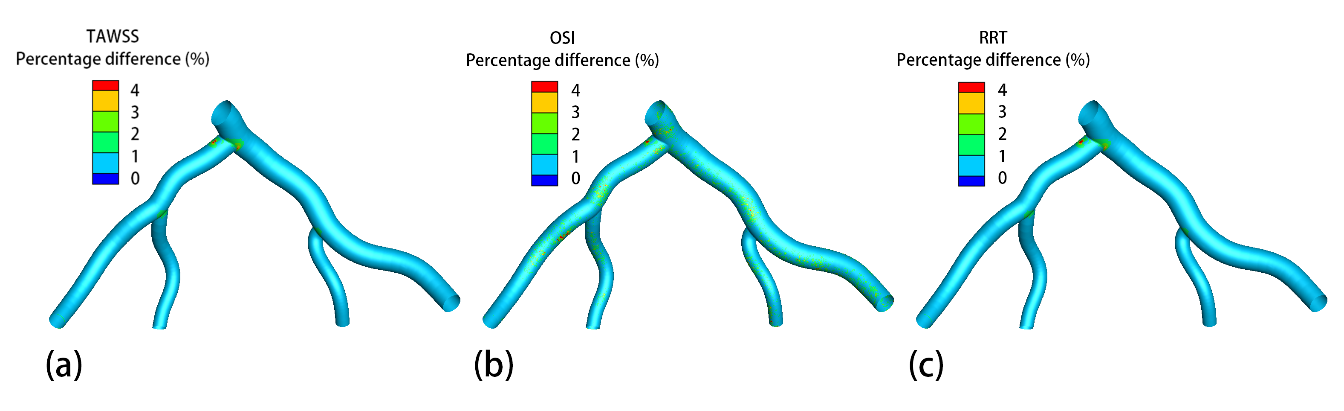


Figure S1. The absolute values of the percentage difference for the parameters $TAWSS$, $OSI$, and $RRT$ between using MESH1 and MESH2. $Percentage difference=\left| \left( {Parameter}_{MESH2}-{Parameter}_{MESH1} \right)/{{Parameter}_{MESH1}} \right|*100\%$

**References**

1 Olgac, U., Kurtcuoglu, V. & Poulikakos, D. Computational modeling of coupled blood-wall mass transport of LDL: effects of local wall shear stress. *Am J Physiol Heart Circ Physiol* **294**, H909-919, doi:10.1152/ajpheart.01082.2007 (2008).

2 Jesionek, K. & Kostur, M. *The Giant Low-density Lipoproteins (LDL) Accumulation in the Multi-layer Artery Wall Model*. (2014).

3 Chung, S. & Vafai, K. Effect of the fluid-structure interactions on low-density lipoprotein transport within a multi-layered arterial wall. *J Biomech* **45**, 371-381, doi:10.1016/j.jbiomech.2011.10.002 (2012).

4 Chung, S. & Vafai, K. Mechanobiology of low-density lipoprotein transport within an arterial wall--impact of hyperthermia and coupling effects. *J Biomech* **47**, 137-147, doi:10.1016/j.jbiomech.2013.09.030 (2014).

5 Huang, Y., Rumschitzki, D., Chien, S. & Weinbaum, S. A fiber matrix model for the growth of macromolecular leakage spots in the arterial intima. *J Biomech Eng* **116**, 430-445 (1994).

6 Chien, S. Molecular and mechanical bases of focal lipid accumulation in arterial wall. *Progress in Biophysics and Molecular Biology* **83**, 131-151, doi:10.1016/s0079-6107(03)00053-1 (2003).

7 Tarbell, J. M. Mass transport in arteries and the localization of atherosclerosis. *Annual review of biomedical engineering* **5**, 79-118 (2003).

8 Cancel, L. M. & Tarbell, J. M. The role of mitosis in LDL transport through cultured endothelial cell monolayers. *Am J Physiol Heart Circ Physiol* **300**, H769-776, doi:10.1152/ajpheart.00445.2010 (2011).

9 Cancel, L. M. & Tarbell, J. M. The role of apoptosis in LDL transport through cultured endothelial cell monolayers. *Atherosclerosis* **208**, 335-341, doi:10.1016/j.atherosclerosis.2009.07.051 (2010).

10 Cancel, L. M., Fitting, A. & Tarbell, J. M. In vitro study of LDL transport under pressurized (convective) conditions. *Am J Physiol Heart Circ Physiol* **293**, H126-132, doi:10.1152/ajpheart.01188.2006 (2007).

11 Ogunrinade, O., Kameya, G. T. & Truskey, G. A. Effect of Fluid Shear Stress on the Permeability of the Arterial Endothelium. *Annals of Biomedical Engineering* **30**, 430-446, doi:10.1114/1.1467924 (2002).

12 Ai, L. & Vafai, K. A coupling model for macromolecule transport in a stenosed arterial wall. *International Journal of Heat and Mass Transfer* **49**, 1568-1591, doi:10.1016/j.ijheatmasstransfer.2005.10.041 (2006).

13 Tedgui, A. & Lever, M. J. Filtration through damaged and undamaged rabbit thoracic aorta. *AJP - Heart and Circulatory Physiology* **247**, H784 (1984).

14 Weinbaum, S., Tzeghai, G., Ganatos, P., Pfeffer, R. & Chien, S. Effect of cell turnover and leaky junctions on arterial macromolecular transport. *The American journal of physiology* **248**, H945-960, doi:10.1152/ajpheart.1985.248.6.H945 (1985).
